# A rare cytogenetically cryptic *MECOM* rearrangement in a patient with myelodysplastic neoplasm and *SF3B1* mutation identified by RNA sequencing: a case report

**DOI:** 10.3389/fonc.2026.1742267

**Published:** 2026-01-19

**Authors:** Ye Jin, Zi-Jun Xu, Chao-Ran Lv, Zhen Qian, Xiang-Mei Wen, Sheng Xiao, Jiang Lin, Jun Qian

**Affiliations:** 1 Department of Hematology, Affiliated People’s Hospital of Jiangsu University, Zhenjiang, Jiangsu, China; 2 Institute of Hematology, Jiangsu University, Zhenjiang, Jiangsu, China; 3 Zhenjiang Clinical Research Center of Hematology, Zhenjiang, Jiangsu, China; 4 Laboratory Center, Affiliated People’s Hospital of Jiangsu University, Zhenjiang, Jiangsu, China; 5 Advanced Molecular Pathology Institute of Soochow University and SANO, Suzhou, Jiangsu, China; 6 SANO Medical Laboratories, Suzhou SANO Precision Medicine Ltd., Suzhou, Jiangsu, China

**Keywords:** case report, inv(3)/t(3;3), *MBNL1*, *MECOM* rearrangement, RNA sequencing, *SF3B1* mutation

## Abstract

*MECOM* (the *MDS1* and *EVI1* complex locus) rearrangements have been identified as an independent high-risk factor in acute myeloid leukemia (AML). The diversity of *MECOM* rearrangement partner genes significantly influences disease mechanisms and prognosis. The majority of atypical *MECOM* rearrangements result in *EVI1* overexpression through translocation into super-enhancer-containing regions. This report describes a rare, recurrent *MBNL1*::*MECOM* rearrangement identified in a myelodysplastic neoplasm (MDS) patient with a concurrent *SF3B1* mutation. Although conventional cytogenetics showed a normal karyotype, the rearrangement was confirmed by next-generation sequencing (NGS) and fluorescence *in situ* hybridization (FISH). Concurrently, the patient exhibited high *EVI1* expression, consistent with the common mechanism observed in atypical *MECOM* rearrangements. Given the well-documented association between *SF3B1* mutations and *MECOM* rearrangements, analysis of *MECOM* expression and RNA sequencing (RNA-seq) is crucial for *SF3B1*-mutated patients, even in the absence of elevated blast counts. Furthermore, this case underscores the need for further research into the synergistic biological role of spliceosome mutations and *MECOM* rearrangements in driving leukemia.

## Introduction

Acute myeloid leukemia (AML) with *MECOM* (the *MDS1* and *EVI1* complex locus) rearrangement is recognized as a distinct entity in the 2008 World Health Organization (WHO) classification of hematolymphoid tumors (1). Patients with this subtype exhibit rapid disease progression and poor prognosis, achieving complete remission (CR) rates of only 15-20% with conventional chemotherapy, a median survival time of 4–6 months, and a 5-year overall survival rate of less than 15% (2). The classical *MECOM* rearrangements, derived from inv(3)(q21;q26) or t(3;3)(q21;q26), lead to the *GATA2* enhancer (3q21) adjacent to *MECOM* (3q26), driving *EVI1* overexpression and monoallelic expression of *GATA2* (3). *EVI1*, first identified in retrovirus-induced murine AML models, plays a crucial role in hematopoiesis and leukemogenesis. Its aberrant overexpression is frequently observed in myeloid malignancies, including AML (4). Functionally, *EVI1* regulates erythroid, granulocytic, and megakaryocytic differentiation. Studies demonstrate that it contributes to hematopoietic neoplasia through transcriptional repression, epigenetic dysregulation, and interactions with pathways such as TGF-β/SMAD and PI3K/AKT (5). *EVI1* exists normally in the form of *MDS1*-*EVI1* and is lowly expressed in normal bone marrow. Rearrangements often disrupt the region between exon 1 of *MDS1* and exon 1 of *EVI1*, leading to activated transcription of *EVI1* (6, 7). Beyond the classical inv(3)/t(3;3), a series of atypical *MECOM* rearrangements have been identified. Chromosomal 3q26 translocations in these AML cases frequently involve super-enhancer hijacking genes active in myeloid development (e.g., *CD164*, *PROM1*, *CDK6*, *MYC*), thereby driving *EVI1* overexpression and potential *GATA2* involvement (8, 9). These patients also showed poor treatment response, with *EVI1* overexpression but no or low *MDS1-EVI1* levels (8, 9). Consequently, the latest WHO classification revises this subtype as AML with *MECOM* rearrangement even if peripheral blood and/or bone marrow blasts < 20% (10). To date, more than 30 additional 3q26 rearrangements have been identified (11). Here, we report a new cytopenic case with an *MBNL1*::*MECOM* rearrangement detected by next-generation sequencing (NGS).

## Case report

**Figure 1:**
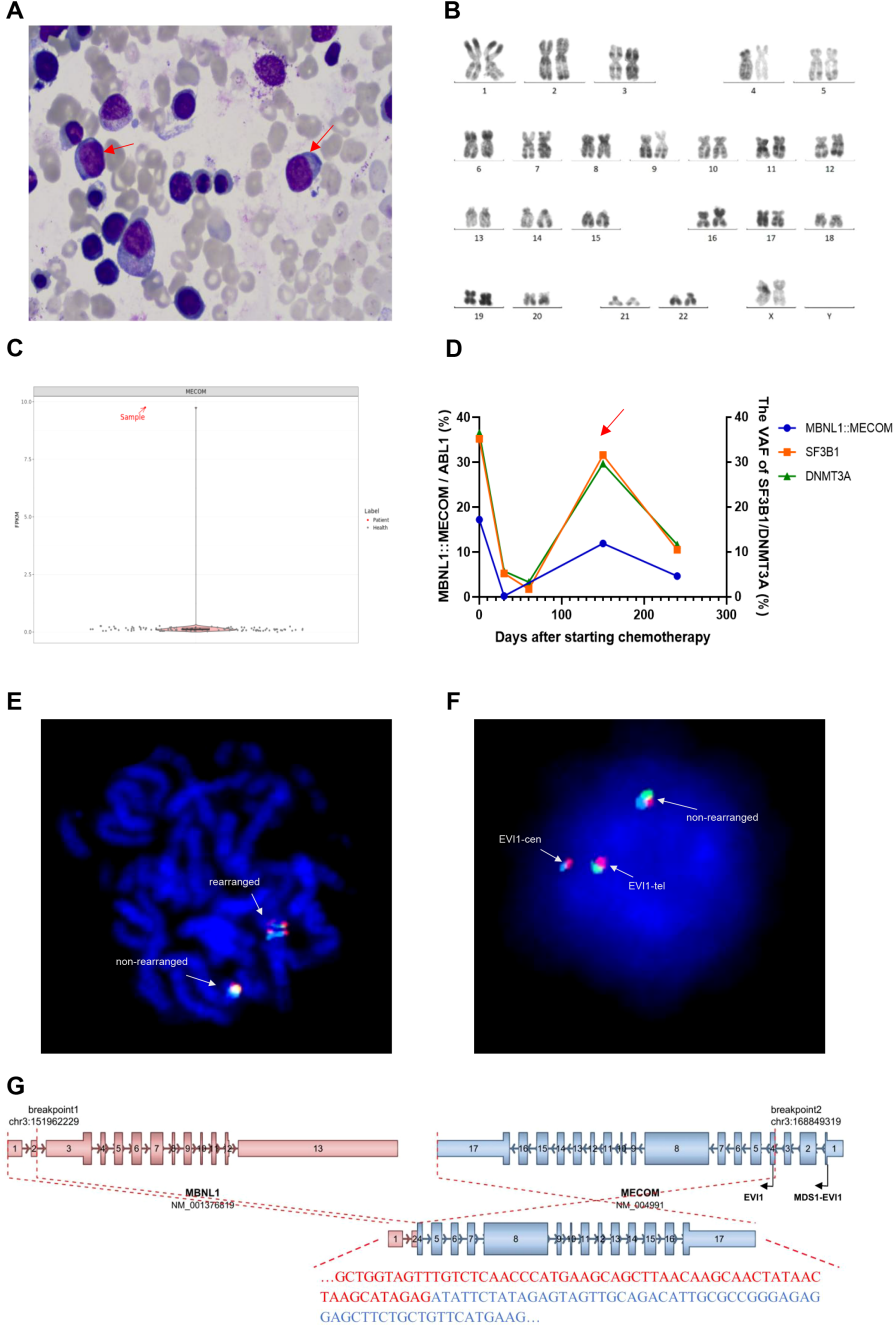
**(A)** Wright-Giemsa staining of the bone marrow aspirate smear showed 3% myeloblasts (indicated by red arrows; ×1000 magnification). The percentage of erythroid cells was increased to 64% with dysplastic features, including megaloblastoid changes, scattered trinucleated erythroblasts, and internuclear bridging. Ring sideroblasts accounted for 28%. According to the World Health Organization (WHO) classification, these findings support a diagnosis of myelodysplastic syndrome with ring sideroblasts (MDS-RS). **(B)** R-banded karyotype analysis of the bone marrow specimen demonstrating a normal karyotype. **(C)** Comparison of *MECOM* expression levels between the patient and the healthy controls. **(D)** Minimal residual disease (MRD) levels were monitored in the patient’s bone marrow samples, quantifying the *MBNL1*::*MECOM* fusion transcript by Real-time Quantitative PCR (RQ-PCR) (*ABL1* as the reference gene) and the *SF3B1* and *DNMT3A* mutations by droplet digital PCR (ddPCR). At the time point indicated by the red arrow, the MRD levels showed a significant increase compared to preceding measurements, indicating the relapse of acute myeloid leukemia (AML). **(E, F)** Fluorescence *in situ* hybridization (FISH) was performed on the patient’s bone marrow sample using a tri-color break-apart *MECOM* (*EVI1*) probe (Kanglu Biotech Co., Ltd.). The blue, green and red probes label the centromeric region, the telomeric region, and the *MECOM* (*EVI1*) locus respectively. FISH confirmed *MECOM* rearrangement in both metaphase **(E)** and interphase **(F)** nuclei. This abnormal pattern was detected in 18% of the scored interphase cells. **(G)** Schematic diagram illustrating the structure and predicted functional consequence of the *MBNL1*::*MECOM* fusion. The breakpoints are located between exons 2 and 3 of *MBNL1* and between exons 3 and 4 of *MECOM*. This disrupts the translation of the full-length *MDS1*-*EVI1* transcript and permits translation initiation from the native *EVI1* start codon, indicating that the fusion transcript does not encode a novel chimeric protein. Similar to other atypical *MECOM* rearrangements, the *MBNL1*::*MECOM* fusion likely drives leukemogenesis by hijacking the *MBNL1* promoter to cause aberrant *EVI1* overexpression.

A 60-year-old woman was admitted to the Affiliated People’s Hospital of Jiangsu University with progressive fatigue over three months. Physical examination revealed sternal tenderness and scattered purpura on the dorsolateral aspect of the right hand. Splenomegaly was present, palpable 6 cm below the costal margin, with a spleen volume of 268.68 cm^3^ measured by color Doppler ultrasonography. Hepatomegaly and lymphadenopathy were not detected. Peripheral blood examination demonstrated pancytopenia: white blood cell 2.32 × 10^9^/L, hemoglobin 8.1 g/dL, and platelet count 18 × 10^9^/L with 1% blasts, 3% promyelocytes and 2% normoblasts. Bone marrow smear showed 3% myeloblasts (**Figure 1A**). The percentage of erythroid cells increased to 64% accompanying megaloblastoid changes, scattered trinuclear erythrocytoblasts and internuclear bridging. Ring sideroblasts increased to 28%. Hypercellularity was found by bone marrow biopsy in which blasts were revealed as 2%~3% by CD34 immunohistochemistry. Cytogenetic analysis demonstrated a normal karyotype (**Figure 1B**). NGS on 318 genes associated with hematologic malignancies identified *SF3B1* R625L mutation with 35.2% of variant allele frequency (VAF) and *DNMT3A* S894Efs27 mutation (VAF 36.5%). Whole-transcriptome RNA sequencing (RNA-seq) of bone marrow sample identified a rare *MBNL1*::*MECOM* fusion gene, accompanied by upregulated *EVI1* expression (**Figure 1C**). Thus, this patient was diagnosed with AML with *MECOM* rearrangement according to the latest WHO classification (10, 11). Following two cycles of azacitidine (AZA) and venetoclax (VEN) therapy, the patient achieved complete remission with incomplete hematologic recovery (CRi). The *MBNL1*::*MECOM* transcript level decreased to 0.22%, and the VAFs of *SF3B1* and *DNMT3A* mutations were both reduced to approximately 5% (**Figure 1D**). After four additional months of AZA+VEN treatment, the *MBNL1*::*MECOM* transcript level increased to 11.93%, and the VAFs of *SF3B1* and *DNMT3A* mutations rose to around 30%, despite the absence of increased blast counts in peripheral blood or bone marrow. Persistent pancytopenia was present also the doses of AZA and VEN were reduced and deferred. The patient is still alive dependently on red blood cell transfusion.

## Discussion

This is the third reported case harboring *MBNL1*::*MECOM* rearrangement. The two previously reported cases include one with chronic myeloid leukemia (CML) in blast crisis (BC) phase (CML-BC) and one with AML secondary to myelodysplastic neoplasm (MDS) (9, 12). Both cases were identified only by NGS, as fluorescence *in situ* hybridization (FISH) was not performed for these two patients. Moreover, detailed clinical data were not described either. This recurrent fusion of *MBNL1*::*MECOM* is derived from the juxtaposition of *MBNL1* gene located on chromosome 3q25.1 and *MECOM*. The subtle inversion between 3q25.1 and 3q26.2 is difficult to detect by conventional karyotype analysis due to the adjacency of these two bands. The disease of this patient would have been misdiagnosed as MDS with *SF3B1* mutation had RNA-seq not been performed, underscoring the critical role of RNA-seq in identifying such cryptic fusions. FISH assay, using the three-color break-apart probes spanning the *MECOM* gene, revealed a break-apart signal pattern in 18% of cells (**Figures 1E, F**), which confirms the *MECOM* rearrangement. The breakpoints were located between exons 2 and 3 of *MBNL1* and between exons 3 and 4 of *MECOM* (**Figure 1G**). Analysis of the spliced sequence using the Expasy Translate tool (https://web.expasy.org/translate/) indicated that the fusion transcript does not encode a novel chimeric protein. Instead, it disrupts the translation of the full-length *MDS1*-*EVI1* transcript, permitting translation initiation from the native *EVI1* start codon (c.665–667 ATG, p.M189), effectively utilizing the *MBNL1* promoter. Consistent with this mechanism, *EVI1* overexpression was detected (**Figure 1C**). Therefore, we propose that similar to other atypical *MECOM* rearrangements, the *MBNL1*::*MECOM* fusion likely drives leukemogenesis by hijacking the *MBNL1* promoter to cause aberrant *EVI1* overexpression. It’s a pity that this study lacks direct functional evidence, such as assay for transposase-accessible chromatin with sequencing (ATAC-seq) or luciferase reporter assays, to conclusively prove that the *MBNL1* promoter directly drives *EVI1* expression in this context. Future experimental validation is required to clearly determine the mechanistic basis of this fusion.

In AML, the recurrent *SF3B1* mutations are most frequently found in the subtypes of myelodysplasia-related AML (MDS-MR) (11). Several previous studies have shown that *SF3B1* mutation is one of the most frequent co-mutations in myeloid neoplasms (9, 13–15). An outstanding biological question is the clonal evolutionary relationship between these two events. A recent study has further confirmed the strong association of *SF3B1* mutation with *MECOM* rearrangements in AML (16). Among 41 AML patients with *SF3B1* mutations, *GATA2*::*MECOM* was found in 10 cases (24%). In their 2 patients, the VAFs of *SF3B1* mutation (both approximately 40%) and the percentages of *MECOM* rearrangement (both approximately 80%) suggest in the prior MDS stage that they exist in the same leukemic clone. In our patient, the proportion of cells harboring the *SF3B1* mutation (35.2% VAF, corresponding to approximately 70.4% of cells) was significantly higher than the proportion of cells with the *MECOM* rearrangement (only 18% as detected by FISH). This finding suggests that the *MBNL1::MECOM* rearrangement may be a secondary event. Although the *MBNL1::MECOM* transcript level was dynamically monitored, concurrent FISH analysis was not performed at each time point. Consequently, two independent clones cannot be excluded definitely. Future studies involving serial sampling to track both the VAF and the percentage of rearranged cells, or to perform single-cell sequencing, are required to ultimately establish the relevance and time sequence of these two clones, thereby providing deeper mechanistic insights into disease progression.

*SF3B1* mutation enhances the leukemogenicity of hematopoietic cells in inv(3) mice (14). Although the coexistence of *SF3B1* mutation does not affect the prognosis of AML patients (17), elucidating the potential synergistic mechanisms between these two events is crucial, given the strong association between *SF3B1* mutation and *MECOM* rearrangement as well as the adverse prognosis associated with *MECOM* rearrangement. These two abnormalities likely jointly exacerbate erythroid differentiation blockade and may form a self-amplifying positive feedback loop, thereby synergistically driving the initiation and progression of leukemia. First, both are closely associated with erythroid differentiation blockade. The *SF3B1* mutation disrupts erythropoiesis by inducing mis-splicing of genes involved in ineffective erythropoiesis (18) and by causing premature downregulation of *GATA1*, which impairs terminal erythroid differentiation and leads to anemia (19). In contrast, *EVI1* overexpression blocks normal erythropoiesis through its interaction with the transcription factor *GATA1* (20). Second, the *SF3B1* mutation can lead to aberrant splicing of *EVI1* itself and accelerate leukemia development *in vivo (*14). The convergence of these mechanisms may have contributed to the aggressive disease biology in this case, which suggests a promising direction for future basic research.

The asynchronous changes of gene mutations, *MECOM* transcript and blasts suggest that *SF3B1* mutation *MECOM* rearrangement are the successive events at preleukemic stage. However, *MECOM* FISH at relapse was not performed to compare the size of *MECOM* rearranged clone with blast percentage, unable to completely rule out the relevance of *MECOM* rearrangement with blasts if the percentage of positive FISH signals is also low.

**Figure 2:**
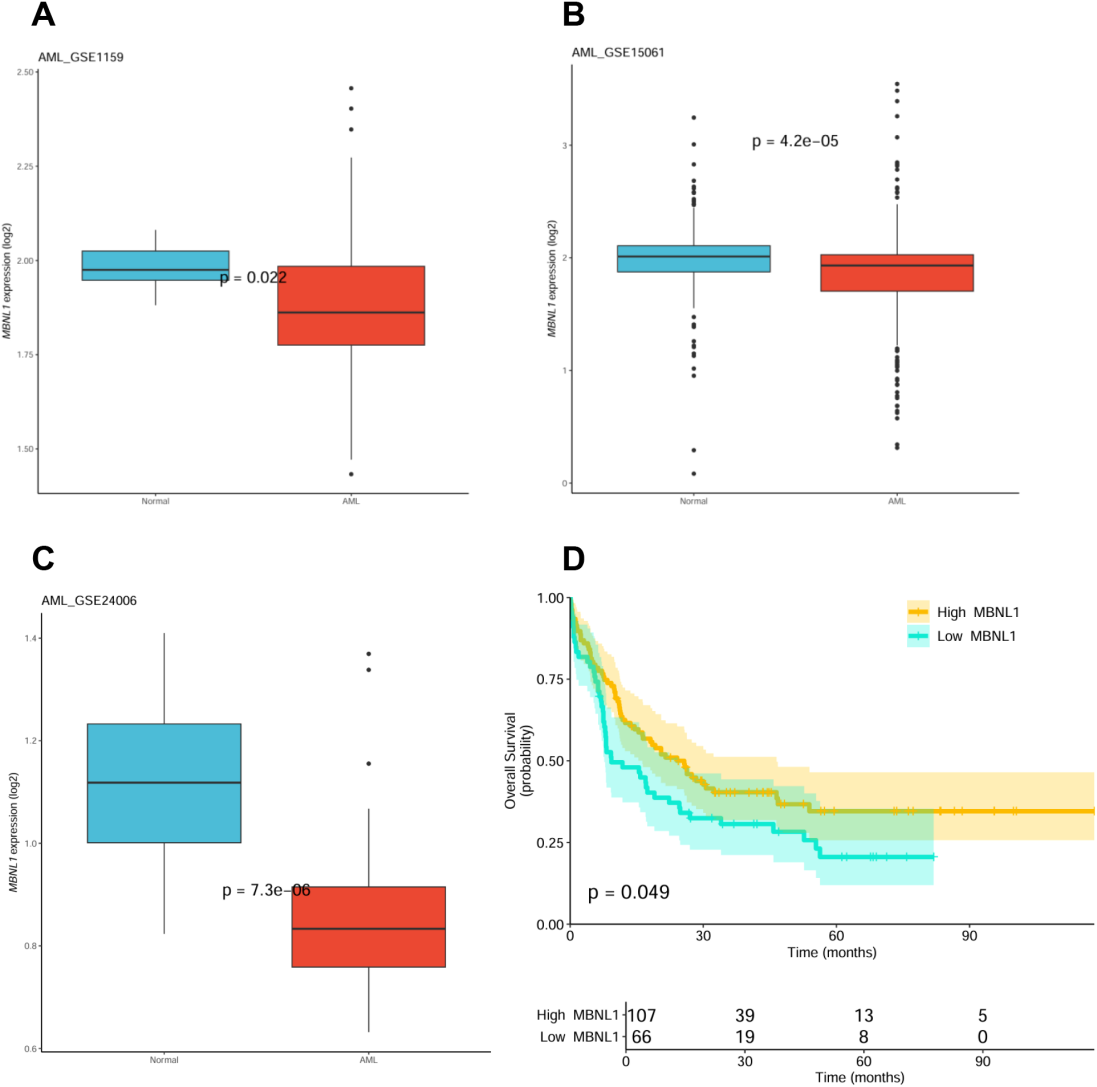
**(A-C)** Analysis of *MBNL1* expression across three independent public datasets. Gene expression data from GSE1159 **(A)**, GSE15061 **(B)**, and GSE24006 **(C)** were analyzed separately. Box plots show *MBNL1* expression in AML bone marrow samples compared to healthy donor (HD) controls, revealing significant downregulation of *MBNL1* in AML. Data were normalized and log2-transformed within each dataset. Due to the independent nature of these datasets and the exploratory purpose of this cross-validation, no cross-dataset batch effect correction was applied. **(D)** Low *MBNL1* expression is associated with inferior overall survival in AML. The Kaplan-Meier survival curve was generated from The Cancer Genome Atlas (TCGA) AML cohort (n=173). Patients were stratified by the median expression level of *MBNL1*. The log-rank *p*-value of 0.049 suggests a trend toward inferior prognosis with low *MBNL1* expression in this univariate model.

Analysis of *MBNL1* expression across AML datasets (GSE1159, GSE15061, GSE24006) reveals its significant downregulation compared to controls (**Figures 2A-C**), and The Cancer Genome Atlas (TCGA) data associates low *MBNL1* expression with poor prognosis (**Figure 2D**). *MBNL1* functions as a tumor suppressor and RNA metabolism regulator (21, 22). Its disruption via rearrangement, coupled with *SF3B1* mutation, could synergistically exacerbate splicing errors, providing a compounded proliferative advantage to leukemic cells (23).

## Conclusion

In summary, we reported a rare recurrent *MBNL1*::*MECOM* rearrangement derived from inv(3)(q25.1; q26.2) in a cytopenic patient, accompanying *SF3B1* mutation. Given the adverse prognosis associated with *MECOM* rearrangements, it is crucial to actively search for them in *SF3B1*-mutated patients using appropriate FISH or RNA-seq, even in the absence of cytogenetic clues or excessive blasts. As a single case report with inherent limitations and relatively short follow-up to date, we will continue monitoring this patient’s disease course. Furthermore, we anticipate that additional cases of *MBNL1*::*MECOM* or other rare *MECOM* partner rearrangements will be identified and reported, enhancing our understanding of this high-risk AML subgroup. Meanwhile, this case underscores the need for further research into the synergistic biological role of spliceosome mutations and *MECOM* rearrangements in driving leukemia.

## Data Availability

The original contributions presented in the study are included in the article/supplementary material. Further inquiries can be directed to the corresponding authors.

## References

[1] Swerdlow SH Campo E Harris NL Jaffe ES Pileri SA Stein H . WHO Classification of Tumours of Haematopoietic and Lymphoid Tissues. 4th. Lyon: IARC Press (2008).

[2] Gröschel S Lugthart S Schlenk RF Valk PJ Eiwen K Goudswaard C . High EVI1 expression predicts outcome in younger adult patients with acute myeloid leukemia and is associated with distinct cytogenetic abnormalities. J Clin Oncol. (2010) 28:2101–7. doi: 10.1200/JCO.2009.26.0646, PMID: 20308656

[3] Gröschel S Sanders MA Hoogenboezem R de Wit E Bouwman BAM Erpelinck C . A single oncogenic enhancer rearrangement causes concomitant EVI1 and GATA2 deregulation in leukemia. Cell. (2014) 157:369–81. doi: 10.1016/j.cell.2014.02.019, PMID: 24703711

[4] Vriend J Delwel R Pastoors D . Mechanisms of enhancer-driven oncogene activation. Int J Cancer. (2026) 158:333–41. doi: 10.1002/ijc.35330, PMID: 39853740 PMC12628035

[5] Liang B Wang J . EVI1 in leukemia and solid tumors. Cancers (Basel). (2020) 12:2667. doi: 10.3390/cancers12092667, PMID: 32962037 PMC7564095

[6] Soderholm J Kobayashi H Mathieu C Rowley JD Nucifora G . The leukemia-associated gene MDS1/EVI1 is a new type of GATA-binding transactivator. Leukemia. (1997) 11:352–8. doi: 10.1038/sj.leu.2400584, PMID: 9067573

[7] Hinai AA Valk PJM . Review: Aberrant EVI1 expression in acute myeloid leukaemia. Br J Haematol. (2016) 172:870–8. doi: 10.1111/bjh.13898, PMID: 26729571

[8] Ottema S Mulet-Lazaro R Beverloo HB Erpelinck C van Herk S van der Helm R . Atypical 3q26/MECOM rearrangements genocopy inv(3)/t(3;3) in acute myeloid leukemia. Blood. (2020) 136:224–34. doi: 10.1182/blood.2019003701, PMID: 32219447

[9] Gao J Gurbuxani S Zak T Kocherginsky M Ji P Wehbe F . Comparison of myeloid neoplasms with nonclassic 3q26.2/MECOM versus classic inv(3)/t(3;3) rearrangements reveals diverse clinicopathologic features, genetic profiles, and molecular mechanisms of MECOM activation. Genes Chromosomes Cancer. (2022) 61:71–80. doi: 10.1002/gcc.23004, PMID: 34668265

[10] Khoury JD Solary E Abla O Akkari Y Alaggio R Apperley JF . The 5th edition of the world health organization classification of haematolymphoid tumours: myeloid and histiocytic/dendritic neoplasms. Leukemia. (2022) 36:1703–19. doi: 10.1038/s41375-022-01613-1, PMID: 35732831 PMC9252913

[11] Swerdlow SH Campo E Harris NL Jaffe ES Pileri SA Stein H . WHO Classification of Tumours of Haematopoietic and Lymphoid Tissues. 5th. Lyon: International Agency for Research on Cancer (2022).

[12] Branford S Wang P Yeung DT Thomson D Purins A Wadham C . Integrative genomic analysis reveals cancer-associated mutations at diagnosis of CML in patients with high-risk disease. Blood. (2018) 132:948–61. doi: 10.1182/blood-2018-02-832253, PMID: 29967129

[13] Lavallée VP Gendron P Lemieux S D’Angelo G Hébert J Sauvageau G . EVI1-rearranged acute myeloid leukemias are characterized by distinct molecular alterations. Blood. (2015) 125:140–3. doi: 10.1182/blood-2014-07-591529, PMID: 25331116 PMC4358966

[14] Tanaka A Nakano TA Nomura M Yamazaki H Bewersdorf JP Mulet-Lazaro R . Aberrant EVI1 splicing contributes to EVI1-rearranged leukemia. Blood. (2022) 140:875–88. doi: 10.1182/blood.2021015325, PMID: 35709354 PMC9412007

[15] Gröschel S Sanders MA Hoogenboezem R Zeilemaker A Havermans M Erpelinck C . Mutational spectrum of myeloid Malignancies with inv(3)/t(3;3) reveals a predominant involvement of RAS/RTK signaling pathways. Blood. (2015) 125:133–9. doi: 10.1182/blood-2014-07-591461, PMID: 25381062 PMC4334729

[16] Huber S Haferlach T Meggendorfer M Hutter S Hoermann G Baer C . SF3B1 mutations in AML are strongly associated with MECOM rearrangements and may be indicative of an MDS pre-phase. Leukemia. (2022) 36:2927–30. doi: 10.1038/s41375-022-01734-7, PMID: 36271152 PMC9712091

[17] McCarter JGW Nemirovsky D Famulare CA Farnoud N Mohanty AS Stone-Molloy ZS . Interaction between myelodysplasia-related gene mutations and ontogeny in acute myeloid leukemia. Blood Adv. (2023) 7:5000–13. doi: 10.1182/bloodadvances.2023009675, PMID: 37142255 PMC10471939

[18] Clough CA Pangallo J Sarchi M Ilagan JO North K Bergantinos R . Coordinated missplicing of TMEM14C and ABCB7 causes ring sideroblast formation in SF3B1-mutant myelodysplastic syndrome. Blood. (2022) 139:2038–49. doi: 10.1182/blood.2021012652, PMID: 34861039 PMC8972092

[19] Lieu YK Liu Z Ali AM Wei X Penson A Zhang J . SF3B1 mutant-induced missplicing of MAP3K7 causes anemia in myelodysplastic syndromes. Proc Natl Acad Sci U.S.A. (2022) 119:e2111703119. doi: 10.1073/pnas.2111703119, PMID: 34930825 PMC8740767

[20] Laricchia-Robbio L Premanand K Rinaldi CR Nucifora G . EVI1 Impairs myelopoiesis by deregulation of PU.1 function. Cancer Res. (2009) 69:1633–42. doi: 10.1158/0008-5472.CAN-08-2562, PMID: 19208846

[21] Taylor K Sznajder LJ Cywoniuk P Thomas JD Swanson MS Sobczak K . MBNL splicing activity depends on RNA binding site structural context. Nucleic Acids Res. (2018) 46:9119–33. doi: 10.1093/nar/gky565, PMID: 29955876 PMC6158504

[22] Zhang Q Wu Y Chen J Tan F Mou J Du Z . The regulatory role of both MBNL1 and MBNL1-AS1 in several common cancers. Curr Pharm Des. (2022) 28:581–5. doi: 10.2174/1381612827666210830110732, PMID: 34459372

[23] Tabaglio T Low DH Teo WKL Goy PA Cywoniuk P Wollmann H . MBNL1 alternative splicing isoforms play opposing roles in cancer. Life Sci Alliance. (2018) 1:e201800157. doi: 10.26508/lsa.201800157, PMID: 30456384 PMC6238595

